# Establishment of a humanized animal model of systemic sclerosis in which T helper-17 cells from patients with systemic sclerosis infiltrate and cause fibrosis in the lungs and skin

**DOI:** 10.1038/s12276-022-00860-7

**Published:** 2022-09-29

**Authors:** Min-Jung Park, Youngjae Park, Jeong Won Choi, Jin-Ah Baek, Ha Yeon Jeong, Hyun Sik Na, Young-Mee Moon, Mi-La Cho, Sung-Hwan Park

**Affiliations:** 1 The Rheumatism Research Center, Catholic Research Institute of Medical Science, College of Medicine, The Catholic University of Korea, Seoul, Republic of Korea; 2 Division of Rheumatology, Department of Internal Medicine, Seoul St. Mary’s Hospital, College of Medicine, The Catholic University of Korea, Seoul, Republic of Korea; 3 Department of Biomedicine & Health Sciences, College of Medicine, The Catholic University of Korea, 222, Banpo-daero, Seocho-gu, Seoul, 06591 Republic of Korea; 4 Department of Medical Life Sciences, College of Medicine, The Catholic University of Korea, 222, Banpo-daero, Seocho-gu, Seoul, 06591 Republic of Korea

**Keywords:** Autoimmunity, Autoimmune diseases

## Abstract

Systemic sclerosis (SSc) is a chronic autoimmune disease characterized by inflammation, microangiopathy, and progressive fibrosis in the skin and internal organs. To evaluate the pathophysiologic mechanisms and efficacies of potential therapeutics for SSc, a preclinical model recapitulating the disease phenotypes is needed. Here, we introduce a novel animal model for SSc using immunodeficient mice injected with peripheral blood mononuclear cells (PBMCs) from SSc patients. Human PBMCs acquired from SSc patients and healthy controls were transferred into NOD.Cg-*Prkdc*^*scid*^*Il2rg*^*tm1Wjl*^ (NSG) mice with concurrent bleomycin injection. Blood, skin, and lung tissues were acquired and analyzed after PBMC engraftment. In addition, we investigated whether the humanized murine model could be used to assess the efficacy of potential therapeutics for SSc. Human PBMCs from SSc patients and healthy controls were engrafted into the blood, skin, and lung tissues of NSG mice. Histological analysis of affected tissues from mice treated with SSc PBMCs (SSc hu-mice) demonstrated substantial inflammation, fibrosis and vasculopathy with human immune cell infiltration and increased expression of IL-17, TGF-β, CCL2, CCL3, and CXCL9. The proportions of circulating and tissue-infiltrating T helper 17 (Th17) cells were elevated in SSc hu-mice. These cells showed increased expression of CXCR3 and phosphorylated STAT3. SSc hu-mice treated with rebamipide and other potential Th17-cell-modulating drugs presented significantly reduced tissue fibrosis. Mice injected with patient-derived PBMCs show promise as an animal model of SSc.

## Systemic sclerosis: Humanized mouse model to boost research

A humanized mouse model of the autoimmune disease systemic sclerosis (SSc) could improve understanding of disease progression and provide a trial platform for potential treatments. SSc results in inflammation and progressive fibrosis in the skin, heart, lungs and kidneys. Existing animal models for SSc are unable to fully mimic the mechanisms behind the disease. Mi-La Cho and Sung-Hwan Park at the Catholic University of Korea, Seoul, South Korea, and co-workers injected peripheral blood cells from patients with SSc into immune-deficent mice, generating a humanized animal model. Several weeks after, the team analysed blood and tissue samples from the mice and found significant inflammation and fibrosis in the skin and lungs, consistent with SSc. Levels of proinflammatory proteins and specific human T-helper cells were significantly elevated, providing possible insights into disease initiation and progression.

## Introduction

Systemic sclerosis (SSc) is a chronic autoimmune disease characterized by inflammation, microvasculopathy, and progressive fibrosis in multiple organs^1^. Although skin involvement is one of the most prominent phenotypes, other organs, such as the heart, lungs, and kidneys, can also be affected, and their involvement is a prognostic factor for SSc^1,2^. There is no curative treatment for SSc^1,2^. Because SSc is rare and its clinical manifestations are heterogeneous, an animal model recapitulating SSc features is needed to investigate the pathophysiologic mechanisms and evaluate the efficacy of potential therapeutics.

Various animal models for SSc have been introduced. Injecting exogenous bleomycin^3^ or transplanting bone marrow into laboratory mice^4^ induces skin fibrosis or graft-versus-host disease (GVHD) resembling SSc. Rodents with mutations in genes related to signals causing fibrosis, such as friend leukemia integration 1 (Fli1)-deficient^5^ or fos-related antigen 2 (Fra-2) transgenic (Tg) mice^6,7^, as well as naturally occurring tight skin 2 (Tsk2) mice^8^, are used as animal models. The injection of type V collagen, a potential autoantigen in SSc, may induce SSc-like features^9^. However, most animal models have a limited ability to recapitulate the phenotypes and pathophysiologic mechanisms of SSc^10^.

In this context, humanized animal models using immunodeficient mice engrafted with cells or tissues from diseased patients are novel preclinical models for various pathologic conditions, including autoimmune diseases^11^. By applying these genetically modified immuno-susceptible murine systems, which are amenable to xenografting, we can directly implant human immune cells^12^. In the pathogenesis of SSc, several types of immune cells—including CD4 + and CD8 + T cells and B cells—are recruited by activated endothelial cells and induce the differentiation of myofibroblasts, which is responsible for fibrosis in SSc^13,14^. A recent report supports the potential of such humanized mouse models engrafted with human dendritic cells as preclinical models of SSc^15^.

We developed a novel preclinical animal model for SSc using immunodeficient NOD.Cg-*Prkdc*^*scid*^*Il2rg*^*tm1Wjl*^ (NSG) mice^16^ and injecting them with peripheral blood mononuclear cells (PBMCs) from patients with SSc. This murine model recapitulated the key pathologic features of SSc—inflammation, fibrosis, and vasculopathy. Human immune cells were engrafted in mice, and activated T helper 17 (Th17) cells, a subset of CD4 + T cells, were increased in engrafted PBMCs and significantly infiltrated murine tissues. Chemokines, such as CCL2, CCL3, and CXCL9, recruited human immune cells to affected organs. We also evaluated the potential of the humanized mouse model for assessing the efficacy of candidate therapeutics for SSc.

## Materials and methods

### SSc patients and the isolation of PBMCs

Peripheral blood was acquired from healthy controls (HC, *n* = 7) and SSc patients (*n* = 13) who visited Seoul St. Mary’s Hospital, a tertiary referral hospital in the Republic of Korea. All SSc patients fulfilled the 2013 classification criteria^17^ for SSc. The demographic and clinical features of the participants are listed in Supplementary Table 1. PBMCs were isolated from heparinized blood by Ficoll-Paque density gradient centrifugation^18^.

### Mice and the induction of a humanized model

Six- to eight-week-old female NSG mice obtained from the Jackson Laboratory (Bar Harbor, ME, USA) were used. The mice were maintained under specific-pathogen-free conditions in an animal facility and given autoclaved food and water. A HEPA filter system was used to exclude bacteria and viruses from the air in the facility.

To develop a humanized model, freshly isolated human PBMCs from HCs or SSc patients were intravenously injected into NSG mice (5 × 10^6^ cells per mouse). After 2 weeks, NSG mice were injected with bleomycin (0.25 mg/mL) subcutaneously every day for 5 weeks. To test the therapeutic efficacy of rebamipide, 3 weeks after the induction of SSc in mice, recipient mice were administered rebamipide (100 mg/kg) every day for 4 weeks via oral feeding. Control mice were administered vehicle (dimethyl sulfoxide diluted in saline) in the same manner as the treatment group.

### Flow cytometry and the measurement of autoantibodies

Mononuclear cells from the peripheral blood and spleens of humanized mice and PBMCs from patients were reacted with fluorescent antibodies (BD Biosciences, San Diego, CA, USA) against CD4 (clone RPA-T4), CD8 (clone SK1), CD19 (clone HIB19), CD25 (clone M-A251), CD14 (clone MφP9), IL-17 (clone SCPL1362), phosphorylated STAT3 (Tyr705, pSTAT3, clone 4/P-STAT3), and collagen type I (COL1A1, R&D Systems, Minneapolis, Minn). Prior to intracellular staining, the cells were restimulated for 4 h with phorbol myristate acetate (25 ng/mL, Sigma-Aldrich) and ionomycin (250 ng/mL, Sigma-Aldrich) in the presence of GolgiSTOP (BD Biosciences, San Diego, CA, USA). Intracellular staining was performed using a kit (eBioscience, Thermo Fisher Scientific, Waltham, MA, USA) following the manufacturer’s protocol. Flow cytometry was performed using a FACSCalibur instrument (BD Biosciences, San Diego, CA, USA). Mouse antibodies targeting human endothelin receptor A (ETAR) were measured using a commercial enzyme-linked immunosorbent assay kit (MyBioSource Inc., San Diego, CA, USA). Detailed measuring protocols followed the ref. ^19^.

### Histological assessment

Harvested tissue was fixed in 10% (v/v) neutral-buffered formalin and embedded in paraffin. Sections (5 µm thick) were stained with hematoxylin and eosin (H&E). Stained tissue sections were examined using a photomicroscope (Olympus, Tokyo, Japan) (×100 or ×200). Dermal thickness was measured with IMT iSolution Lite software (version 10.0, IMT iSolution Inc., Canada). Masson’s trichrome (MT) staining was conducted using a ready-to-use kit (Masson’s Trichrome Stain Kit, 25088-1, Polysciences, Inc., Warrington, PA). MT-stained slides were observed for changes in collagen deposition using a photomicroscope (Olympus, Tokyo, Japan) (×100). Lung fibrosis was assessed by a grading system^20^: 0, normal lung; 1, minimal fibrous thickening of alveolar or bronchiolar vessels; 3, moderate thickening of walls without obvious damage to lung architecture; 5, increased fibrosis with definite damage to lung structure and the formation of fibrous bands or small fibrous masses; 7, severe distortion of lung structure and large fibrous areas; and 8, total fibrous obliteration of the field. Skin inflammation was scored as follows^21^: 0, none; 2, focal infiltrates; and 4, widespread infiltrates. Lung inflammation was scored as follows^22^: 0, no inflammatory cells; 1, <10%; 2, 10–25%; 3, 25–50%; and 4, >50%. The severity of vasculopathy was scored as follows, defined as averaged scores of multiple components^23^ (neutrophil infiltration, fibrinoid necrosis and deposits, leukocytoclasis, and endothelial swelling): 0, absent; 1, mild; 2, moderate; and 3, severe. Histological analysis was performed by three experimenters in a blinded manner.

### Immunohistochemistry

Immunohistochemistry was performed using VECTASTAIN ABC kits (Vector Laboratories, Burlingame, CA, USA). Sections were incubated with primary antibodies against CD4 (rabbit polyclonal, Abcam, Inc., Cambridge, UK), CD8 (rabbit polyclonal, Abcam, Inc., Cambridge, UK), CD19 (rabbit polyclonal, Abcam, Inc., Cambridge, UK), TGF-β (mouse monoclonal TB21, Invitrogen, Carlsbad, CA, USA), IL-17 (mouse monoclonal 41809, R&D Systems, Minneapolis, Minn), STAT3 (BD Bioscience, San Jose, CA, USA), pSTAT3 (Tyr705, BD Bioscience, San Jose, CA, USA), α-smooth muscle actin (α-SMA, mouse monoclonal ACTA2 [4A8-2H3], Novus Biologicals, Centennial, CO, USA), VEGF (rabbit polyclonal, Santa Cruz, CA, USA), CTGF (rabbit polyclonal), Novus Biologicals, Centennial, CO, USA, caspase-3 (rabbit polyclonal, Abcam, Inc., Cambridge, UK), endothelin-1 (rabbit polyclonal, Abcam, Inc., Cambridge, UK), and chemokine ligands (CCL2 [rabbit polyclonal, Abcam, Inc., Cambridge, UK], CCL3 [goat polyclonal, Invitrogen, Carlsbad, CA, USA], and CXCL9 [rabbit polyclonal, Abcam, Inc., Cambridge, UK]) overnight at 4 °C. The primary antibody was detected with a biotinylated secondary linking antibody, followed by incubation with a streptavidin-peroxidase complex for 1 h. The final color was produced with DAB chromogen (Dako, Carpinteria, CA, USA). Positive cells, which were identified by a dark brown deposit in the nucleus, were enumerated in 10 randomly selected high-power fields (HPF, ×400, 2.37 mm^2^). The sections were counterstained with H&E and photographed using a photomicroscope (Olympus, Tokyo, Japan).

### Confocal microscopy

Skin tissue sections (7 µm thick) were fixed in 4% (v/v) paraformaldehyde and stained using fluorescein isothiocyanate (FITC)-, phycoerythrin (PE)-, PerCP-Cy5.5-, or allophycocyanin-conjugated monoclonal antibodies against human CD4 (clone M-T477, BD Bioscience, San Jose, CA, USA), IL-17 (clone SCPL1362, BD Bioscience, San Jose, CA, USA), CXCR3 (clone G025H7, BD Bioscience, San Jose, CA, USA), pSTAT3 (Tyr705, clone 4/P-STAT3, BD Bioscience, San Jose, CA, USA), procollagen type 1 (clone M-58, Abcam, Inc., Cambridge, UK) and DAPI (Invitrogen, Waltham, MA, USA). After incubation overnight at 4 °C, stained sections were visualized by confocal microscopy (LSM 510 Meta, Zeiss, Göttingen, Germany). Positive cells were counted, and the counts are expressed as the means ± SEMs.

### Statistical analysis

Statistical analysis was performed using Prism (version 9.2, GraphPad Software Inc., San Diego, CA). The significance of differences was determined by *t* tests for two groups and one-way analysis of variance for three groups. Statistical significance was determined at *p* < 0.05.

## Results

### Human immune cells engrafted in the blood and tissues of immunodeficient mice

**Fig. 1:**
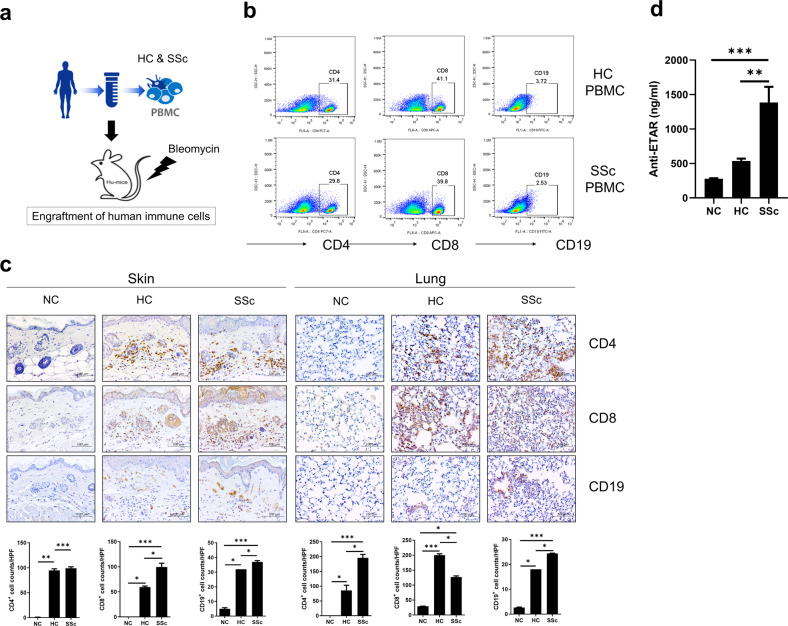
Development of humanized murine model of systemic sclerosis (SSc) using human peripheral blood mononuclear cells (PBMCs). **a** Schematic representation of the humanized mouse model of SSc. PBMCs isolated from blood samples of SSc patients and healthy controls (HCs) were injected intravenously into NSG mice (5 × 10^6^ cells per mouse). At 2 weeks after PBMC injections, bleomycin was injected subcutaneously into mice every day for 5 weeks. **b** Six weeks after the initial induction, peripheral blood samples of recipient mice were collected and analyzed for the level of engraftment of human CD4+, CD8 + T cells and CD19 + B cells by flow cytometry. **c** Representative immunohistochemistry (IHC) images showing human CD4+, CD8 + T cells and CD19 + B cells infiltrating the skin and lung tissues of recipient mice (upper panels). **d** Serum levels of anti-human endothelin receptor A (ETAR) antibodies in PBMC-treated mice determined by ELISA. Original magnification ×400. Scale bars represent 100 μm. Cell counts per high-power field (HPF) of each immune cell subset are shown in the lower panels. Data are presented as the means ± SEMs. NC, nontreated control mice. *p < 0.05; **p < 0.01; ***p < 0.001.

NSG mice present multiple mutations in the common gamma chain of the IL-2 receptor and protein kinase DNA-activated catalytic polypeptide, thereby bearing severe deficiencies in innate and adaptive immunity^11^. After the isolation of PBMCs from the blood of SSc patients and HCs, we intravenously injected human cells into NSG mice (Fig. 1a). Next, low doses of bleomycin were injected to induce inflammation. Because spontaneous GVHD occurs approximately 6 to 7 weeks after the time of PBMC injection^11^, sampling was performed 6 weeks after induction. Human PBMCs from SSc patients and HCs were engrafted and identified using anti-human CD4 + and CD8 + T-cell and CD19 + B-cell antibodies in blood from recipient mice (Fig. 1b). Engrafted human CD4 + and CD8 + T cells and B cells infiltrated the skin and lung tissues of mice, which are the organs typically affected by SSc (Fig. 1c). T and B cells from SSc patients were present in skin and lung tissues significantly more frequently than those from HCs (Fig. 1c). In addition, the activities of engrafted autoreactive B cells were determined by measuring significantly increased serum levels of anti-human ETAR antibodies in mice treated with PBMCs from SSc patients (Fig. 1d).

### Engraftment of SSc PBMCs induces inflammation, fibrosis and vasculopathy in skin and lung tissues

**Fig. 2:**
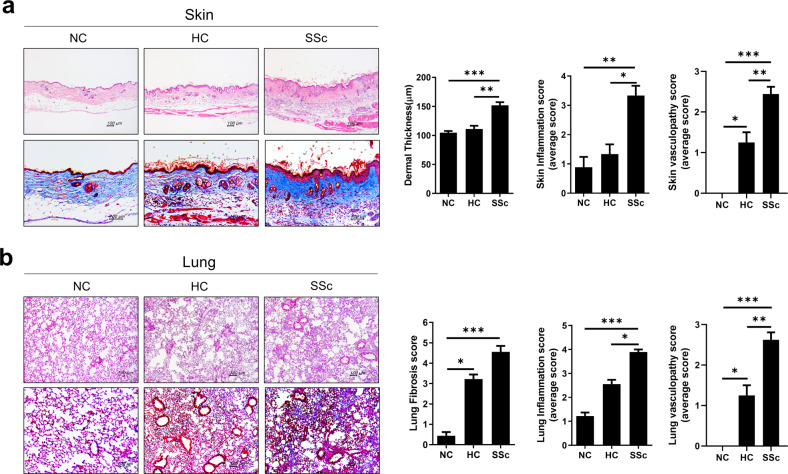
Engraftment of SSc PBMCs induces inflammation, fibrosis, and vasculopathy in the skin and lung tissues of immunodeficient mice. **a**, **b** At 45 days after the injection of human PBMCs (Fig. 1), skin and lung tissues were harvested from humanized mice. **a** Representative images of mouse skin tissues stained with hematoxylin and eosin (H&E) and Masson’s trichrome (MT) are shown in the left panels. Dermal thickness, inflammation and vasculitis scores are shown in the right panels. **b** Representative images of mouse lung tissues stained with H&E and MT are shown in the left panels. Fibrosis, inflammation and vasculitis scores are shown in the right panels. Original magnification ×100. Scale bars represent 100 μm. Data are presented as the means ± SEMs. NC nontreated control mice, HC HC-engrafted mice, SSc SSc-engrafted mice. *p < 0.05; **p < 0.01; ***p < 0.001.

Because local inflammation, fibrosis, and vasculopathy are major pathologic components in SSc-affected organs^1^, we determined the degree of inflammation and fibrosis in skin and lung tissues by staining for collagen and scoring inflammation and vasculopathy in each organ^20,21,23^. Dermal thickness, inflammation and vasculopathy scores were significantly higher in NSG mice injected with SSc PBMCs (SSc hu-mice) than in those injected with HC PBMCs (HC hu-mice), whereas there were no differences between nontreated mice and HC hu-mice (Fig. 2a). In lung tissues, HC hu-mice showed higher fibrosis scores than nontreated mice, but SSc hu-mice showed more severe fibrosis than the other two groups (Fig. 2b). The inflammation and vasculopathy scores of lung tissues presented similar tendencies to the fibrosis scores.

### Expression of inflammatory, profibrotic, and endothelial cell activation markers is increased in the skin and lung tissues of SSc-humanized mice

**Fig. 3:**
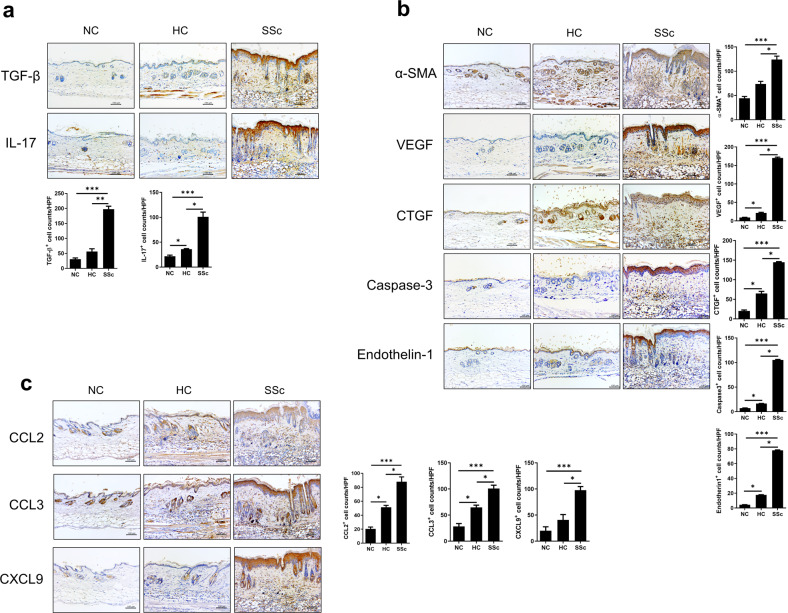
The expression levels of inflammatory, profibrotic, and endothelial cell activation markers are increased in skin tissues from humanized mice. Six weeks after SSc (Fig. 1), skin tissues were harvested and analyzed by IHC for specific markers. **a** Representative images of IHC (upper panels) and graphs (lower panels) using antibodies against inflammatory markers (TGF-β and IL-17). **b** Representative images of IHC (left panels) and graphs (right panels) using antibodies for fibrosis and endothelial cell activation markers (α-SMA, VEGF, CTGF, caspase-3, and endothelin-1). **c** Representative images of IHC (left panels) and graphs (right panels) using antibodies against chemokines (CCL2, CCL3, and CXCL9). Original magnification ×200. Scale bars represent 100 μm. Bars represent positive cell counts per HPF, and values are means ± SEMs. **p < 0.01; ***p < 0.001.

We assessed the expression level of soluble mediators, including cytokines and chemokines, associated with SSc pathologies in the skin and lung tissues of humanized mice 6 weeks after induction. TGF-β is the key cytokine in the differentiation of myofibroblasts^24^, which are implicated in SSc and are highly conserved in humans and mice^25^. IL-17 mediates SSc pathogenesis by influencing fibroblast activation and proliferation^26^. Therefore, we detected the expression levels of TGF-β and IL-17 in dermal tissues by immunochemistry. The expression levels of both cytokines were significantly elevated in SSc hu-mice (Fig. 3a).

We also determined the expression levels of factors related to fibrosis and vasculopathy. CTGF is crucial in myofibroblast-mediated fibrosis^27^^,^ and α-SMA is a marker of activated myofibroblasts^28^. The expression levels of both factors were significantly increased in skin tissues from SSc hu-mice compared to those from HC hu-mice or nontreated mice (Fig. 3b). Vasculopathy is reflected by the activation and apoptosis of endothelial cells^1^. VEGF, endothelin-1, and caspase-3 are markers of endothelial dysfunction resulting in vasculopathy^9^. In dermal tissues, these markers were significantly increased in SSc hu-mice, indicating more severe endothelial cell activation (Fig. 3b).

Chemokines play important roles in the pathologic changes in SSc^29^. Activated fibroblasts and endothelial cells release CCL2, thereby recruiting monocytes and inducing the production of extracellular matrix^30^. In addition to CCL2, the expression of CCL3 is increased in the skin of SSc patients^31^. Monocytes produce other chemokines, such as CXCL9 and CXCL10, in conjunction with dendritic cells^32,33^. The release of these chemokines leads to the infiltration of T cells expressing the receptor CXCR3^34^. According to immunohistochemical staining for these proinflammatory chemokines, the expression levels of CCL2, CCL3, and CXCL9 were significantly higher in dermal tissues of SSc hu-mice than those of other mice (Fig. 3c).

**Fig. 4:**
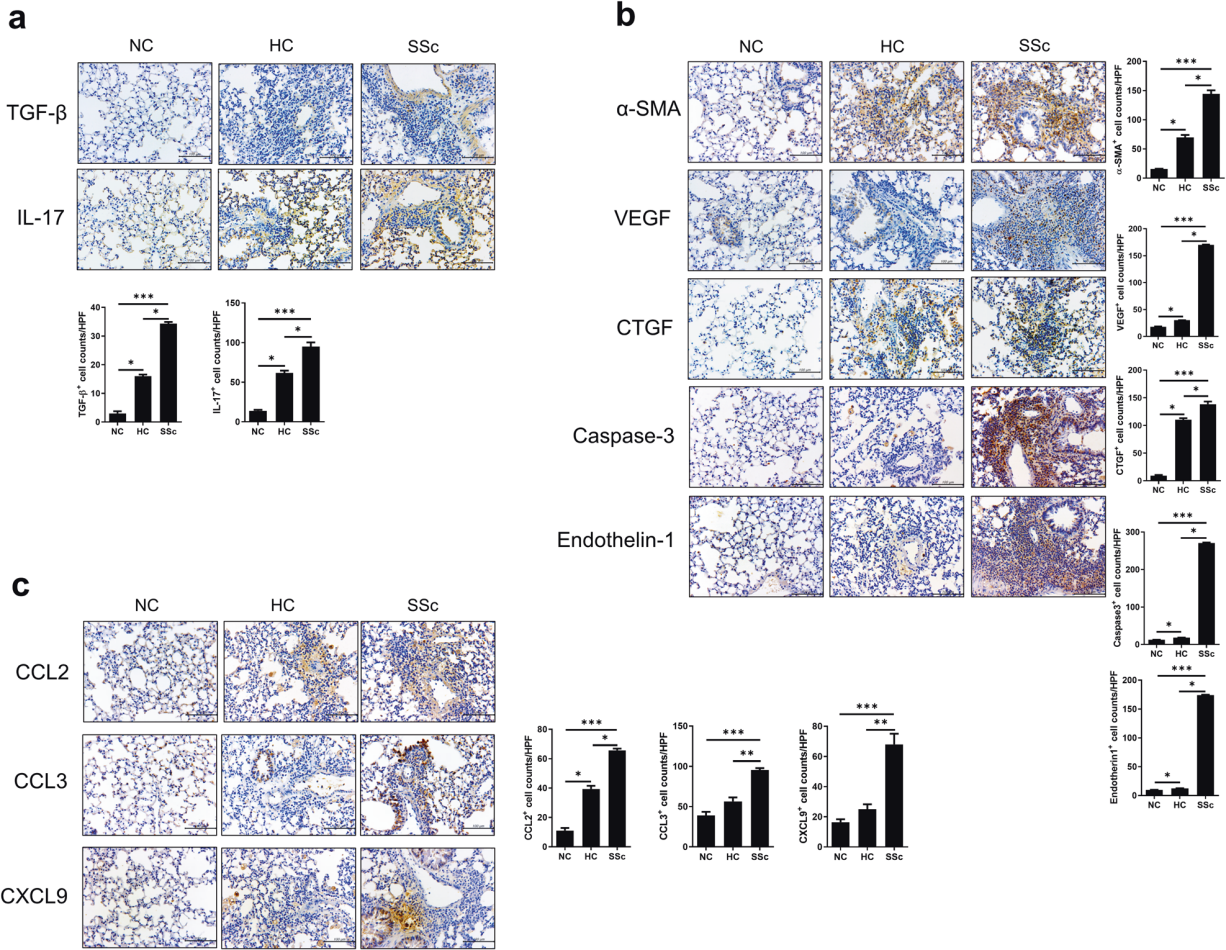
The expression levels of inflammatory, profibrotic, and endothelial cell activation markers are increased in lung tissues from humanized mice. Six weeks after SSc induction (Fig. 1), lung tissues were harvested and analyzed by IHC for specific markers. **a** Representative images of IHC (upper panels) and graphs (lower panels) using antibodies against inflammatory markers (TGF-β and IL-17). **b** Representative images of IHC (left panels) and graphs (right panels) using antibodies for fibrosis and endothelial cell activation markers (α-SMA, VEGF, CTGF, caspase-3, and endothelin-1). **c** Representative images of IHC (left panels) and graphs (right panels) using antibodies against chemokines (CCL2, CCL3, and CXCL9). Original magnification ×400. Scale bars represent 100 μm. Bars represent positive cell counts per HPF, and values are means ± SEMs. *p < 0.05; **p < 0.01; ***p < 0.001.

We also evaluated the expression levels of SSc-related cytokines and chemokines in lung tissues from humanized mice. The histological findings of lung tissues stained and subjected to immunohistochemistry were similar to those of skin tissues (Fig. 4a–c). The expression levels of proinflammatory and profibrotic soluble factors were higher in lung tissues from SSc hu-mice than in those from nontreated or HC hu-mice. Finally, we also performed immunohistochemical staining for other SSc-related factors, such as IL-4, interferon-gamma, IL-10, and Foxp3. These soluble factors were also significantly increased in skin and lung tissues from SSc hu-mice, except IL-10 in lung tissues (Supplementary Fig. 1).

### Active Th17 cells are increased by SSc PBMCs

**Fig. 5:**
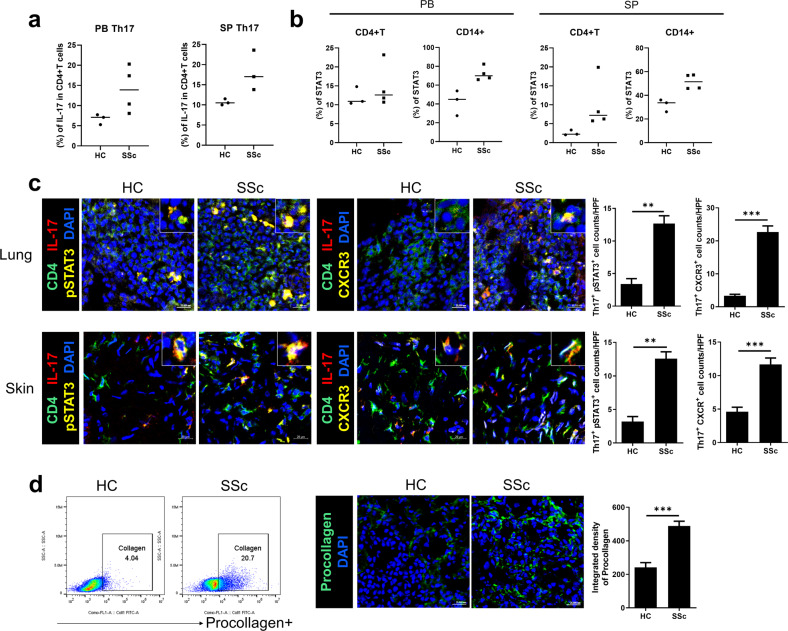
The proportion of T helper 17 (Th17) cells and the expression levels of STAT3 and procollagen are increased in humanized mice by SSc PBMCs. Six weeks after the induction of SSc in mice (Fig. 1), peripheral blood (PB), spleen (SP), skin and lung tissues were acquired. **a** Flow cytometric analysis showing the percentages of human Th17-cell populations in the PB and SP of recipient mice. **b** Flow cytometric analysis showing the percentages of human CD4 + T cells and CD14 + cells positive for pSTAT3 among total CD4 + T cells and CD14 + cells. **c** Representative confocal images showing cells positive for CD4 (green), IL-17 (red), pSTAT3 (yellow), and CXCR3 (yellow) in lung (upper panels) and skin tissues (lower panels). Representative merged images are shown in the right upper corner of each image. The number of positive cells is presented in the right panels. **d** Flow cytometric plots showing procollagen-positive cells in PB (left panels). Representative confocal images showing cells positive for procollagen in lung tissue (middle panels). Integrated densities of procollagen are presented in the right panels. Original magnification ×400. Scale bars represent 20 μm. Bars indicate means. **p < 0.01; ***p < 0.001.

To investigate the disease-inducing mechanism, we evaluated the distribution of subtypes of immune cells potentially related to the pathogenesis of SSc in humanized mice. We collected peripheral blood and spleens from recipient mice 6 weeks after PBMC injection. Despite several inconsistencies, Th17 cells and IL-17 are implicated in the pathogenesis of SSc^35–37^. Because IL-17 induced fibrosis and inflammation in other animal models of SSc^38^, we determined the proportion of Th17 cells in humanized mice. According to flow cytometry, the engrafted human Th17-cell number was marginally higher in the peripheral blood and spleens of SSc hu-mice than in those of HC hu-mice (Fig. 5a).

Elevated STAT3 expression has been reported in PBMCs from SSc patients^39^. Because STAT3 is a pivotal mediator of various inflammatory signals^40^, we quantified engrafted CD4 + and CD14 + cells with intracellular expression of activated STAT3 in peripheral blood and spleens from recipient mice. Flow cytometry showed modestly elevated numbers of pSTAT3-expressing CD4 + and CD14 + cells in peripheral blood and spleens from SSc hu-mice (Fig. 5b).

Next, we investigated whether infiltrating Th17 cells in affected tissues are activated with increased expression of pSTAT3 using confocal microscopy. The number of IL-17-positive CD4 + cells with pSTAT3 expression was higher in skin tissues from SSc hu-mice than in HC hu-mice (Fig. 5c). Furthermore, the number of Th17 cells expressing CXCR3 was significantly higher in dermal sections of SSc hu-mice (Fig. 5c). These results suggest that engrafted Th17 cells infiltrating affected tissues are activated with increased expression of STAT3.

The number of procollagen-positive cells was also increased in SSc hu-mice. Because the expression of procollagen reflects fibrotic activity^41^, we determined the proportion and number of procollagen-expressing cells in peripheral blood and skin tissues of humanized mice by flow cytometry and confocal microscopy, respectively. SSc hu-mice showed a significantly higher number of procollagen-positive cells in peripheral blood and skin than HC hu-mice (Fig. 5d).

### Preventive efficacies of treatment candidates for the development and progression of SSc

**Fig. 6:**
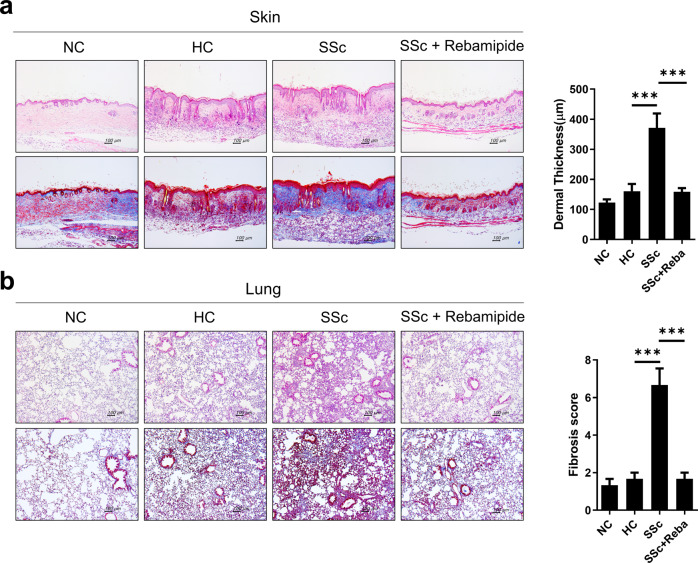
Assessment of the preventive efficacies of rebamipide in a humanized mouse model of SSc. Rebamipide or vehicle was orally administered every day for 4 weeks into humanized mice induced as described in Fig. 1. At 49 days after PBMC engraftment, skin and lung tissues were harvested. **a** Representative images of H&E- and MT-stained skin tissues are shown in the left panels. Dermal thickness is presented in the right panel. **b** Representative images of H&E- and MT-stained lung tissues are shown in the left panels. The fibrosis score is presented in the right panel. Original magnification ×100. Scale bars represent 100 μm. Values are means ± SEMs. ***p < 0.001.

Finally, we evaluated the potential of the humanized murine model for assessing the preventive efficacy of potential therapeutics for SSc development. Rebamipide is a gastroprotective agent with immunomodulatory effects. Rebamipide modulates IL-17 production and Th17-cell differentiation by inhibiting STAT3 signals^42–45^. Considering the results in Fig. 5, we used rebamipide as a potential therapeutic. At 3 weeks after the injection of human PBMCs, vehicle or rebamipide was orally administered for 3 weeks. At 6 weeks after induction, skin and lung tissues were obtained from the mice and assessed. According to histological analysis by H&E and MT staining, the degree of fibrosis in skin and lung tissues was significantly suppressed in rebamipide-treated mice despite SSc PBMC injection (Fig. 6a, b). Rebamipide showed preventive effects on the development and progression of inflammation and fibrosis in the tissues of these humanized mice. Additionally, we also assessed other therapeutic candidates, such as secukinumab, an anti-IL-17 antibody, and tofacitinib, a Janus kinase inhibitor, using this model. These two agents directly target Th17, which accounts for the main cellular mechanism of the humanized model, or inhibit multiple proinflammatory cytokine signals. Similar to rebamipide, secukinumab and tofacitinib reduced inflammation and fibrosis in the lung and skin tissues of SSc hu-mice (Supplementary Fig. 2). These results suggest that humanized mice can be used to assess the efficacy of candidate therapeutics for SSc.

## Discussion

A humanized animal model using immunodeficient mice injected with SSc PBMCs recapitulated the histological phenotypes of SSc-affected skin and lung tissues. The pathologic triad of SSc—inflammation, fibrosis, and vasculopathy—was reproduced by the model. Increased numbers of Th17 cells with STAT3 expression and procollagen-positive cells in blood and tissues induced SSc-like features. Rebamipide, a Th17-cell and STAT3 signal regulator, showed therapeutic efficacy by reducing lung and skin fibrosis in SSc-humanized mice.

NSG mice can be genetically engineered to readily accommodate xenogeneic tissues, organs, or blood from disease-affected participants^11,12,16^. In this study, we transferred the PBMCs of SSc patients, which include immune cells potentially affecting the pathogenesis of SSc, into NSG mice. The lung and skin tissues of NSG mice with PBMCs from SSc patients showed typical pathologic features of SSc in humans. Most animal models for SSc have limitations in recapitulating the key features of SSc^10^. A bleomycin-induced preclinical model for SSc is easy to perform and reproduces tissue fibrosis but not autoimmunity^46^. Mice with genetic overexpression of specific factors related to fibrosis, such as TGF-β or PDGF, enable the analysis of the roles of these factors in SSc, but other pathologies, such as vasculopathy, are absent^46^. SSc PBMC-engrafted mice presented the pathologic triad—fibrosis, inflammation, and vasculopathy—of SSc in affected tissues.

Myofibroblasts induce fibrosis by producing extracellular matrix; various immune cells are involved in autoimmunity and inflammation in SSc^2^. Because PBMCs isolated from SSc-affected participants include possibly disease-causative immune cells, the SSc-mimicking features of the humanized murine system could be due to the engraftment of these cells. Various proinflammatory cytokines and chemokines, which are cross-reactive between human and mouse cells, are assumed to be involved^2,47^. The expression of CCL2, CCL3, CXCL9, and CXCR3, their receptors, was increased in the affected tissues of recipient mice. These chemokines recruit several types of immune cells, such as monocytes and T cells^47^. Therefore, such chemoattractants could promote the infiltration of human immune cells into recipient tissues, thereby promoting inflammation and fibroblast activation.

The expression of IL-17 is also increased in the skin and lung tissues of SSc PBMC-injected mice. IL-17 is mainly produced by Th17 cells, a specific subset of helper T cells, and affects fibroblasts and endothelial cells by promoting inflammation and fibrosis^26,38^. The results of prior studies on the pathogenic roles of IL-17 and Th17 cells in SSc are discrepant^37,48^. In human blood, circulating IL-17 and Th17 cells are increased in SSc^35,36^, whereas an in vivo study using a rodent model and histological analysis of human tissues showed the opposite results^49^, possibly because of the heterogeneity of target populations and experimental settings. The findings of this and our previous studies indicate a close relationship between Th17 cells and SSc pathogenesis. Th17 cells gain effector functions by intracellular signaling using STAT3, which is activated by proinflammatory cytokines^40^. The infiltration of activated human STAT3-expressing Th17 cells suggests a role for Th17 cells in the development of this SSc-mimicking humanized murine model. Furthermore, the suppression of fibrosis by rebamipide, a STAT3 and Th17 regulator^42–45^, supports a role for Th17 cells in the pathogenesis of SSc in the model.

Although our findings suggest the humanized mouse to have promise as a preclinical model for SSc, several concerns need to be investigated. Because the clinical characteristics of patients with SSc are heterogeneous, it should be determined whether consistent development of SSc-mimicking features could be replicated by various human donors. In the present study, no association of the phenotypes of humanized mice with the clinical features of donor patients was observed. The consideration of these respects is crucial for the further application and generalization of this model. In addition, the window for evaluating phenotypes and therapeutic efficacies is narrow due to the development of GVHD, possibly confounding the results. This limits the utility of our humanized mouse model for assessing the chronic features of SSc.

In summary, humanized immunodeficient mice immunized with PBMCs from affected patients recapitulated the phenotypes of SSc. The activated Th17 cells and monocytes with profibrotic properties from SSc patients induced the development of fibrosis. Our humanized mouse model will be useful for preclinical studies of SSc with respect to the roles of immune cells and the efficacies of candidate therapeutics.

## Supplementary information

Supplementary material
