# Supplementary material for: Establishment of a humanized animal model of systemic sclerosis in which T helper-17 cells from patients with systemic sclerosis infiltrate and cause fibrosis in the lungs and skin
Source: Exp Mol Med. 2022 Sep 29;54(9):1577–85. doi: 10.1038/s12276-022-00860-7 (PMC9534900; doi:10.1038/s12276-022-00860-7)
Supplement: Supplementary file 1 — Supplementary material [file 12276_2022_860_MOESM1_ESM.pdf]

Supplementary Table 1. Demographic and clinical features of patients with systemic sclerosis

Subject number	Age (years)	Sex	Duration (years)	Cutaneous type	Autoantibody profiles	Medication status	Organ involvements
1	59	female	6	limited	anti-centromere antibody	glucocorticoid	
2	50	female	14	limited	anti-centromere antibody	methotrexate, glucocorticoid	
3	54	female	25	limited	all negative	azathioprine, glucocorticoid	
4	45	female	2	limited	anti-centromere antibody, anti-Ro antibody	hydroxychloroquine, glucocorticoid	
5	51	female	1	diffuse	anti-scl-70 antibody	methotrexate, glucocorticoid	Interstitial lung disease
6	58	female	3	limited	anti-centromere antibody	glucocorticoid	
7	68	female	3	limited	anti-centromere antibody	glucocorticoid	
8	46	female	2	limited	all negative	methotrexate, azathioprine, glucocorticoid	Interstitial lung disease
9	70	female	27	diffuse	anti-scl-70 antibody, anti-ribosomal P antibody	azathioprine, glucocorticoid	digital ulcer
10	50	female	23	diffuse	anti-scl-70 antibody	cyclophosphamide, glucocorticoid	Interstitial lung disease, digital ulcer
11	43	male	5	diffuse	anti-Ro antibody	none	
12	66	female	24	limited	anti-Ro antibody, anti-RNP antibody	methotrexate, glucocorticoid	Interstitial lung disease
13	60	female	23	diffuse	anti-Ro antibody, anti-scl-70 antibody	azathioprine, glucocorticoid	Interstitial lung disease

Supplementary Fig. 1 The expression levels of IL-4, interferon (IFN)-γ, IL-10, and FoxP3 in lung and skin tissues from humanized mice. Six weeks after induction of humanized mice (**Fig. 1**), skin and lung tissues were harvested and analyzed by immunohistochemistry (IHC) for specific markers. Representative images of IHC (upper panels) and graphs (lower panels) using antibodies. Original magnification ×200. Scale bars represent 100 μm. Bars represent positive cell counts per HPF and values are means ± SEM. *, $p < 0.05$; **, $p < 0.01$; ***, $p < 0.001$.

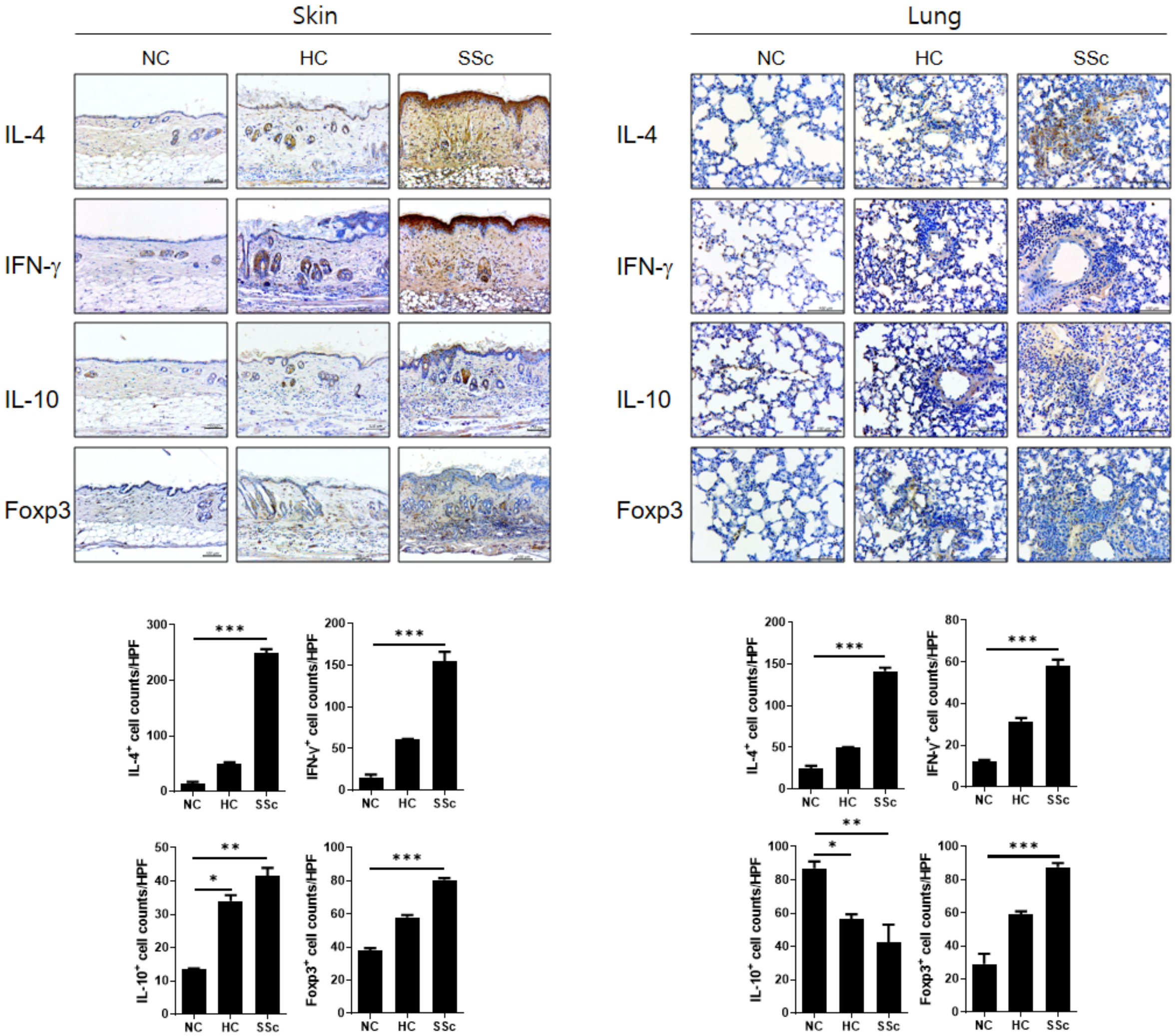
Skin
Lung
NC
HC
SSc
IL-4
IFN-γ
IL-10
Foxp3
IL-4+ cell counts/HPF
IFN-γ+ cell counts/HPF
IL-10+ cell counts/HPF
Foxp3+ cell counts/HPF
***
**
*

Supplementary Fig. 2 Assessment of the therapeutic potential of secukinumab and tofacitinib in a humanized mouse model of SSc. Tofacitinib or vehicle was orally administered every day for 4 weeks into humanized mice induced as described in **Fig. 1**. Secukinumab (10 mg/kg) was subcutaneously injected weekly during the same period. At 49 days after PBMC engraftment, skin and lung tissues were harvested. Representative images of H&E and MT-stained skin (upper panels) and lung (lower panels) tissues are shown in the left pannels. Fibrosis and inflammation scores are presented in the right panel. Original magnification ×100. Scale bars represent 100 μm. Values are means ± SEM. *, $p < 0.05$; **, $p < 0.01$.

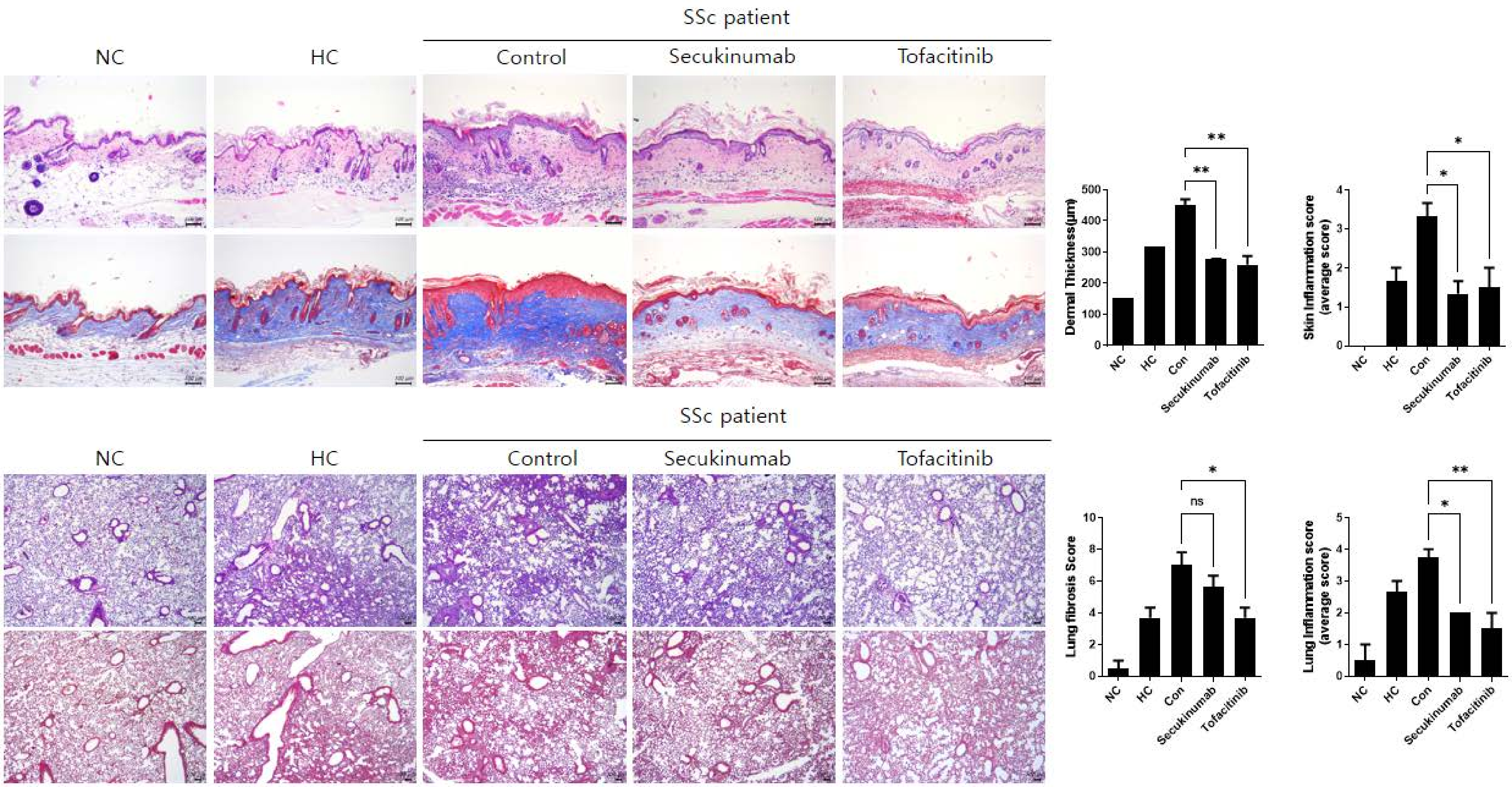

SSc patient
NC
HC
Control
Secukinumab
Tofacitinib
Dermal Thickness(μm)
Skin Inflammation score (average score)
NC
HC
Con
Secukinumab
Tofacitinib
**
*
SSc patient
NC
HC
Control
Secukinumab
Tofacitinib
Lung fibrosis Score
Lung Inflammation score (average score)
ns
*
**
